# Highlights of Strategies Targeting Fibroblasts for Novel Therapies for Rheumatoid Arthritis

**DOI:** 10.3389/fmed.2022.846300

**Published:** 2022-02-17

**Authors:** Cong-Qiu Chu

**Affiliations:** Division of Arthritis and Rheumatic Diseases, Oregon Health & Science University, Section of Rheumatology, VA Portland Health Care System, Portland, OR, United States

**Keywords:** synovial fibroblast, rheumatoid arthritis, fibroblast activation protein, metabolism, proliferation

## Abstract

Synovial fibroblasts of rheumatoid arthritis (RA) play a critical role in perpetuation of chronic inflammation by interaction with immune and inflammatory cells and in cartilage and bone invasion, but current therapies for RA are not directly targeted fibroblasts. Selectively fibroblast targeted therapy has been hampered because of lack of fibroblast specific molecular signature. Recent advancement in technology enabled us to gain insightful information concerning RA synovial fibroblast subpopulations and functions. Exploring fibroblast targeted therapies have been focused on inducing cell death via fibroblast associated proteins; interrupting fibroblast binding to matrix protein; blocking intercellular signaling between fibroblasts and endothelial cells; inhibiting fibroblast proliferation and invasion; promoting cell apoptosis and inducing cellular senescence, and modulating fibroblast glucose metabolism. Translation into clinical studies of these fibroblast targeted strategies is required for evaluation for their clinical application, in particular for combination therapy with current immune component targeted therapies. Here, several strategies of fibroblast targeted therapy are highlighted.

## Introduction

Current therapies for rheumatoid arthritis (RA) have substantially improved the outcome of the disease and the quality of life of these patients. However, achieving and maintaining long-term remission is still challenging. Moreover, a significant proportion of RA patients do not adequately respond to current therapies (1). One of the reasons for the imperfect management of RA is that a critical cell type, fibroblasts, is not adequately targeted.

RA is primarily inflammation of the synovium, cartilage degradation, and bone erosion. In a normal joint, synovium is a thin loosely organized relatively acellular connective tissue without a basal membrane. Instead, synovium is bordered by a lining layer which comprises cells of monocyte in origin (type A synoviocytes) and resident fibroblasts (previously referred as fibroblast-like synoviocytes, type B synoviocytes) (2, 3). The hallmark of RA pathology is hyperplasia of the synovium. In RA, the synovium grows enormously into a mass-like tissue containing a large number of immune and inflammatory cells. In several aspects, RA synovium is considered an analog of a tumor (4). Thus, RA synovium displays neovasculature forming pannus and invades into adjacent cartilage and bone leading to joint destruction (5). The tumor like feature of RA synovium is largely contributed by fibroblasts (4, 6). RA fibroblasts proliferate and are resistant to apoptosis. They build stromal network which harbors immune and inflammatory cells. Moreover, fibroblasts actively interact with immune and inflammatory cells leading to persistent inflammation of the synovium; support the formation of ectopic lymphoid follicles (7). In addition, fibroblasts are effector cells producing inflammatory cytokines participating inflammatory process and matrix metalloproteinase (MMP) and directly invading articular cartilage and subchondral bone. It is well-established that infiltration of immune and inflammatory cells in RA synovium is histologically heterogeneous between individual patients ranging from fully organized lymphoid structures to diffusely distributed lymphoid and myeloid cells throughout the synovium, and to scares of immune and inflammatory cells (pauci-immune). In contrast to the highly variable presence of immune and inflammatory cells, fibroblasts are invariably present in all pathotypes of synovitis (8). Thus, fibroblasts are indispensable in formation of RA synovitis and actively contribute to cartilage and bone destruction. Thereby, a therapeutic strategy directed at modulation of fibroblast function or ablation of fibroblasts has long been proposed (9–12). However, fibroblast targeted therapies have not yet been developed for clinical practice owing to poorly understood molecular mechanisms that drive synovial fibroblast behavior in RA. Recently, more insightful understanding of RA fibroblasts coupled by advanced technology enable us to explore fibroblast targeted therapies for RA. Readers are directed to excellent comprehensive reviews for molecular and cell biology of fibroblasts, their interplay with immune and inflammatory cells in the synovium, and discussion on restoration of synovial homeostasis in RA (12–16). In this article, I shall highlight several different strategies selectively targeting fibroblasts, which have been explored in preclinical arthritis models and/or in early phase of clinical investigations.

## Expansion of Fibroblasts in RA Synovium

The number of fibroblasts in RA synovium increases substantially in both lining and sublining regions. The expansion of fibroblasts is the result of increased proliferation and decreased cell death (17–19). Earlier studies suggest that fibroblasts arise from local epithelial to mesenchymal transition and differentiation from pluripotent mesenchymal stem cells (20–22). There is little evidence indicating *in situ* proliferation of fibroblasts in the lining region. In contrast, recent studies have demonstrated proliferation of sublining fibroblasts (23–25). The gradient reduction of Thy1 (CD90) and augmentation of proteoglycan (PRG)-4 from sublining to lining fibroblasts suggest that divided fibroblasts migrate from sublining to lining region (24, 26). The origin of sublining fibroblasts is not clear. A recent study suggests that circulating preinflammatory mesenchymal (PRIME) cells, which bear hallmarks of synovial sublining fibroblasts, migrate into synovium during RA flare (27). It is conceivable that PRIME cells further differentiate into sublining fibroblasts upon interaction with endothelial cells (15, 26, 27). Several studies have shown that RA fibroblasts display aberrant apoptosis at several levels (28) and also probably due to increased autophagy (17, 19).

## Cell Surface Proteins Serve as Targets for Ablation of Fibroblasts or Modulation of Fibroblast Function

**Figure 1:**
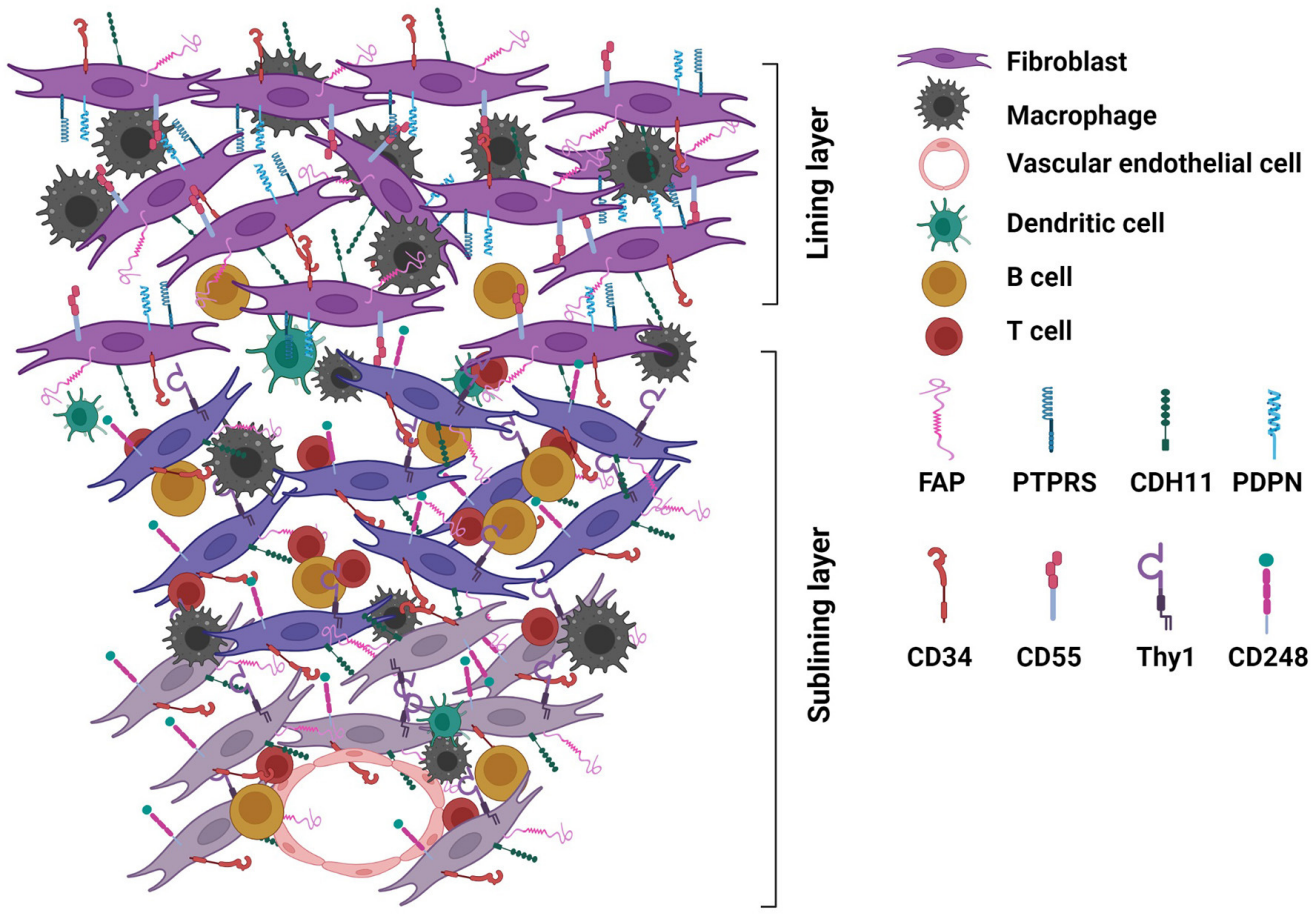
Rheumatoid arthritis (RA) synovium highlighting fibroblasts with surface protein expression. Expansion of fibroblasts substantially contributes to the hyperplasia of RA synovium. Fibroblasts interplay with immune and inflammatory cells to perpetuate inflammation and invade cartilage and subchondral bone leading to joint destruction. Subpopulations of fibroblasts disperse in the lining and sublining layers of the synovium. Distinct profiles of surface proteins expressed by sublining and lining fibroblasts are highlighted here with relevance to fibroblast targeted therapies. Sublining fibroblasts express Thy1 (CD90) and CD248, while lining fibroblasts express CD55, podoplanin (PDPN), and protein tyrosine phosphatase receptor sigma (PTPRS). CD34, cadherin (CDH)-11 and fibroblast activation protein (FAP) are expressed by both sublining and lining fibroblasts [detailed summary of protein expression by different subpopulations of RA synovial fibroblasts is described in Nygaard and Firestein's review (12)].

Several subpopulations of RA synovial fibroblasts have been described based on their distinct profiles of gene and protein expression. It is likely that these subpopulations of fibroblasts function differently, which are related to their locations in the compartments and interaction with other cells in the synovium (Figure 1) (23, 25, 29, 30). The strategy at ablation of fibroblasts has been hampered due to the lack of specific cell surface targets. Recently, several surface proteins have been described for identifying RA synovial fibroblast populations. Namely, CD55, podoplanin, and protein tyrosine phosphatase receptor sigma (PTPRS) are expressed by lining fibroblasts, while Thy1 (CD90), and CD248 are expressed on sublining fibroblasts (Figure 1).

The ideal cell surface targets should be expressed by all subpopulations of fibroblasts. CD34, cadherin-11, and fibroblast activation protein (FAP) can be detected in both lining and sublining fibroblasts (Figure 1) (23–25, 29–32). CD34, expressed by hematopoietic stem cells, is not a specific marker for fibroblasts. CDH11 is critical for lining layer formation and CDH11 deficient mice are resistant to arthritis induction (33); and has been explored as a therapeutic target. However, in a phase II clinical trial, an anti-CDH11 monoclonal antibody failed to show efficacy in RA patients with inadequate response to tumor necrosis factor (TNF) inhibitors (34).

### Fibroblast Activation Protein

FAP is a type II transmembrane protein serving as a serine protease that cleaves the peptide bond between proline and other amino acids. This activity modifies various bioactive molecules (35, 36). FAP is exclusively expressed in fetal cells but not expressed in healthy adult tissue, except bone marrow derived mesenchymal stem cells and wounded tissues (37–40). FAP is best known for its presence in stromal fibroblasts found in over 90% of epithelial tumors (35, 36). FAP expression in tumor cells and stromal fibroblasts may involve tumor invasion since over-expression of FAP in epithelial cells or fibroblasts promoted cell invasion through extracellular matrix (41, 42). In RA synovium, FAP is highly expressed by fibroblasts in the lining layer and the sub-lining layers (4, 7, 29, 43, 44). The expression is highly specific to RA fibroblasts since FAP is expressed in low levels by osteoarthritic and none by normal fibroblasts (43). Similarly, we have demonstrated that FAP is highly expressed in the synovium of arthritic joints in murine models of RA, collagen-induced arthritis (CIA) and arthritis in SKG mice. The pattern of expression is similar to that in RA synovium (7, 43). Importantly, FAP is not expressed in synovium of normal mice. Moreover, levels of FAP expression in CIA joints is correlated with the severity of arthritis. Thus, scanning of FAP expression in the joint has been used to monitor disease activity in CIA (45). The function of FAP expressed by RA fibroblasts is not clear but may be related to the invasion of synovial pannus to cartilage and bone. This notion is supported by the following experimental data. For instance, backcross of FAP gene deficient mice with human TNF transgenic mice lead to amelioration of cartilage damage (46). FAP^+^/Thy1^−^ fibroblasts primarily reside in the lining layer and produce MMP3, MMP9, and MMP13 which are involved in cartilage degradation. In addition, these lining fibroblasts also express CCL9 and TNFSF11, both potently induce osteoclast activity. They also express high levels of receptor activator of nuclear factor kB receptor ligand (RANKL). Whereas, FAP^+^/Thy^+^ fibroblasts mainly reside in the sublining layers are mediating inflammation. These distinct activities of the two subpopulation of fibroblasts were elegantly demonstrated by transfer studies (29). Furthermore, depletion of FAP expressing fibroblasts in serum transfer arthritis model reduced arthritis severity (29). Importantly, FAP gene knockout mice develop normally and have no clinical disease phenotype, suggesting that targeting FAP will be less likely to cause fatal adverse effects (47).

**Figure 2:**
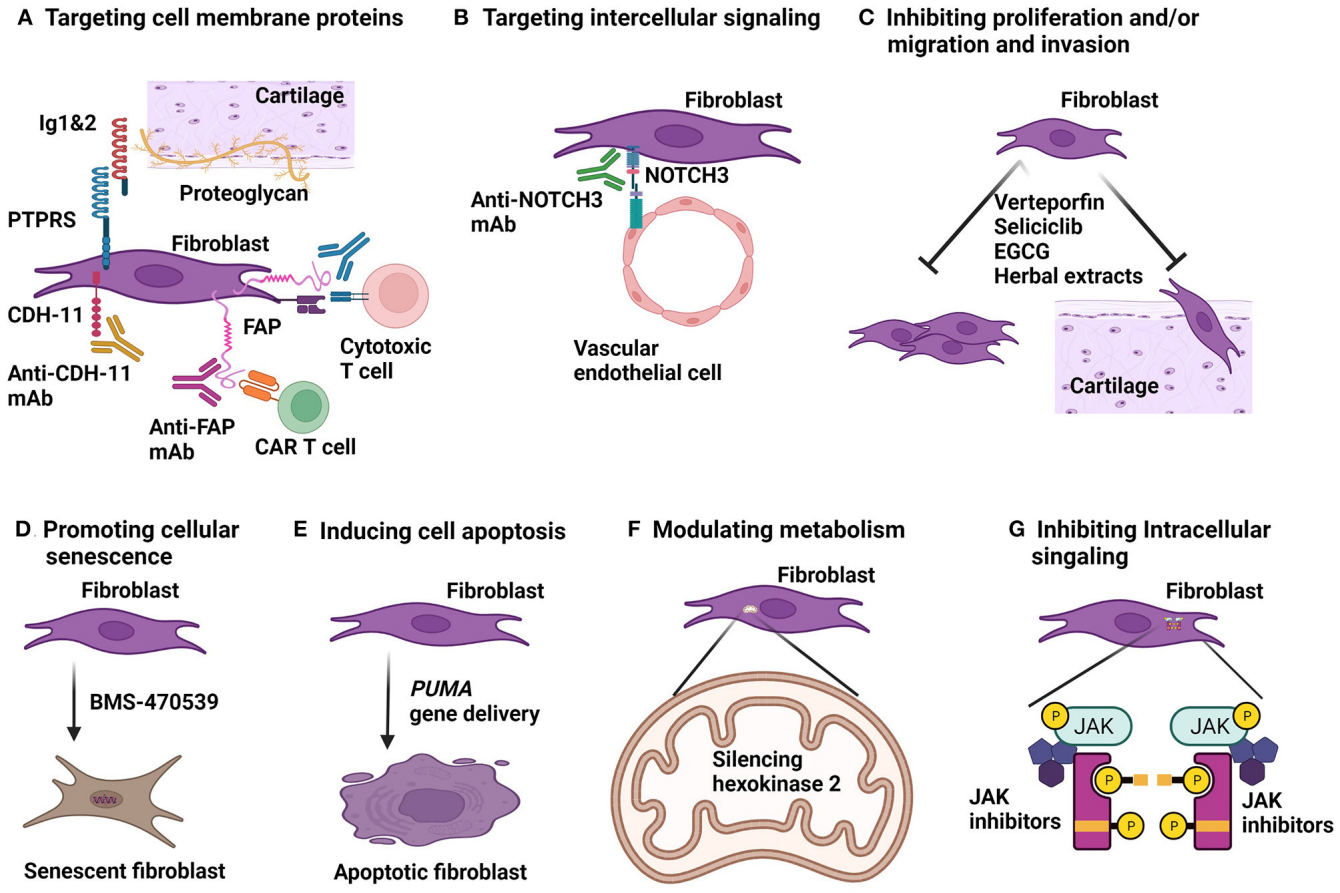
Strategies for fibroblast targeted therapy. **(A)** Cell surface expressed proteins can be targeted in various ways. Therapeutic monoclonal antibodies (mAb) to cadherine-11 (CDH-11) and fibroblast activation protein (FAP) have been developed. FAP can also be targeted by chimeric antigen receptor (CAR) T cells and by vaccination which will provoke antibodies and cytotoxic T cells against FAP *in vivo*. Immunoglobulin like domain 1&2 (Ig1&2) acts as a decoy receptor blocks protein tyrosine phosphatase receptor type S (PTPRS) binding to proteoglycan. **(B)** mAb to neurogenic locus notch homolog protein 3 (NOTCH3) can block interaction between fibroblasts and endothelial cells. **(C)** Several approaches to inhibition of fibroblast proliferation and migration have been investigated. Transcriptional co-activators, Yes-associated protein (YAP) and transcriptional co-activator with PDZ-binding motif (TAZ) are active in RA fibroblasts. Verteporfin blocks YAP/TAZ binding to transcriptional enhanced associate domains (TEAD) and has anti-arthritic effect in arthritis models. Seliciclib blocking RA fibroblast proliferation and has been in a clinical trial for treating RA. Green tea extract, epigallocatechin-3-gallate (EGCG) and several Chinese herbal extracts are shown to inhibit RA synovial fibroblast proliferation, migration and invasion *in vitro* and anti-arthritic effect in animal models of arthritis. **(D)** Activation of a G-protein coupled receptor, melanocortin type 1 receptor (MC1R) by a selective agonist, BMS-470539 can induce senescence and diminish activity of RA fibroblasts. **(E)** Delivery of pro-apoptotic gene, *PUMA* (p53 upregulated modulator of apoptosis) induced RA synovial fibroblast cell death and has anti-arthritic effect in arthritis models. **(F)** Glucose metabolism of RA synovial fibroblasts are high active. Silencing hexokinase 2, a glycolytic enzyme resulted in reduction of migration and invasion of RA fibroblasts *in vitro*. **(G)** Janus kinase (JAK)-signal transducer and activator of transcription (STAT) pathway is active in RA synovial fibroblasts. Fibroblast-selective JAK inhibitors are required to inhibit JAK-STAT mediated cytokine signaling for RA synovial fibroblasts.

Several strategies have been explored in cancer immunotherapy by targeting FAP in tumor models, which can be applied in treating RA. These include monoclonal antibodies, DNA vaccines and chimeric antigen receptor (CAR) T cells against FAP (Figure 2A).

Unfortunately, phase I/II clinical trials with an anti-FAP monoclonal antibody in cancer treatment did not show meaningful clinical efficacy probably due to the fact that the non-toxic unconjugated monoclonal antibody did not induce cell death (48). Recently, Dorst et al. (49) conjugated a near infrared dye, IRDye700DX to a monoclonal antibody (28H1) to FAP and demonstrated that *in vitro* 28H1-700DX was capable of killing FAP expressed fibroblast cell line (3T3) and primary synovial fibroblasts from synovial biopsy of RA patients (50). Furthermore, 28H1-700DX had moderate effect on development of CIA presumably by inducing death of FAP expressing synovial fibroblasts (49). These data indicate the feasibility of anti-FAP monoclonal antibody based therapy for RA is still a viable approach provided that the anti-FAP monoclonal antibody is able to ablate fibroblasts.

DNA vaccines and CAR T cells have been demonstrated to be efficacious for immunotherapy in cancer models and cardiac fibrosis (51–53). Employment of vaccines and CAR T cells to treat RA is distinctive from current RA therapies. Since RA is a chronic disease, it is highly desirable for RA patients to have a treatment which the efficacy will last for a long-term. Vaccination fulfills this requirement. CAR T cells can become memory effector cells (54) and provide continuously surveillance to the joint and generate specific response to FAP to prevent fibroblast expansion. Furthermore, targeted therapies against fibroblasts have the potential to modify disease persistence as opposed to simply inhibiting inflammation. Both vaccination and CAR T cells potentially provide memory immunity against activated fibroblasts by attacking the activation molecule, FAP. These therapies therefore may offer a one-time treatment for a long-term remission. The vaccination approach can also be applied in prevention of RA onset in those at risk population.

Vaccination against FAP has been extensively studied for cancer immunotherapy in animal models. Various strategies have been used. Plasmid cDNA encoding FAP has successfully provoked cellular immune response to FAP and benefit to the host in reducing tumor burden. Since RA is a chronic disease with persistent synovitis which is presumably the result from sustained stimuli. Therefore, a long lasting immunity against FAP will be required to suppress arthritis. Plasmid cDNA injection combined with electroporation which has been successfully applied in tumor models (51) and is being tested in human clinical trials for human papillomaviruses (HPV) (Phase 2b) (55) and Zika virus (Phase 1) (56). The anti-HPV DNA vaccine showed efficacy in treating cervical intraepithelial neoplasia (55); and anti-Zika DNA vaccine elicited anti-Zika virus immune responses (56). It is expected that FAP DNA vaccine will elicit both antibody and T cell immune responses to FAP and arthritis suppression.

Treating solid tumors with FAP CAR T cells alone or in combination with tumor specific antigens have been investigated with success in multiple preclinical tumor models (52, 57, 58). It is expected that CAR T cells against FAP will also display therapeutic effects in arthritis models. Considering that CAR T cells may generate long-lasting memory immune response and one time treatment is needed will justify its application.

Since FAP is an endogenous protein, toxicity or off target effects upon depletion of FAP expressing cells has been a concern. However, data from preclinical studies support this approach is a viable safe therapy. For example, mice with genetic ablation of FAP grow normally without pathological phenotypes (47). Many studies reported successful inhibition of tumor growth but without severe clinical toxicity or impaired wound healing (51, 52, 58–63). In contrast, a recent study with genetic ablation of FAP in BM-MSC showed severe toxicity with anemia and weight loss (59). Explanation for the toxicity include direct suppression of hemopoietic cells and depletion of fat tissue. The findings in this study is in contrast to those with globally targeting FAP expressing cells. These include recently published studies using DNA vaccines (51) and CAR T cells against FAP (52). Both studies have been successfully used in tumor models with efficacy in inhibition of tumor growth without severe toxicity. In particular, there were no cachexia or anemia observed in animals whose FAP expressing cells were depleted. Since FAP is not expressed by normal fibroblasts, depletion of FAP expressing cells does not affect normal fibroblasts. Data from the most recent study confirmed that depletion of FAP expressing fibroblasts in an arthritis model was not toxic (29). Thus, FAP expressing cells were depleted by injection of diphtheria toxin during arthritis in transgenic FAP luciferase diphtheria toxin receptor reporter mice. All these observations support the feasibility and safety for a treatment of arthritis by depleting FAP expressing fibroblasts. The first in human Phase 1 clinical trial with CAR T cells against FAP in mesothelioma patients is reported to be feasible and safe (64). Needless to say, further human studies are required to assess the usage of FAP targeted CAR T cell therapy for non-malignant conditions such as RA.

### Protein Tyrosine Phosphatase Receptor Sigma

Another transmembrane protein, PTPRS is highly enriched in RA and experimental arthritis synovial lining fibroblasts (Figure 1) (31, 32). PTPRS acts like a receptor or ligand binding via N-terminal extracellular immunoglobulin-like domains 1 and 2 (Ig1&2) to extracellular matrix protein, proteoglycan (PG) of various types. Ig1&2 may bind to heparin sulfate (HS) or chondroitin sulfate (CS) glycosaminoglycan (GAG) moieties of PG (65). HS-containing PG competes with CS-containing PG in binding to PTPRS, a mechanism is termed “PG switch” (65). Synovial fibroblast attachment to cartilage is an important process for invasion of cartilage during inflammatory arthritis (66). This attachment is PG dependent and mediated by HS moieties and can be inhibited by exogenous cartilage derived CS (31). This suggests that manipulation of PG switch in arthritic joint can be therapeutic. Furthermore, soluble recombinant Ig1&2 can act as a decoy receptor to inhibit PTPRS expressed by fibroblasts binding to PG, thereby be used to treat arthritis (Figure 2A). Indeed, Ig1&2 decoy receptor suppressed KBxN serum transfer induced arthritis and chronic arthritis in KBxN mice (31). Interestingly, a fusion protein, Fc-Ig1&2 decoy receptor treatment can be combined with TNF inhibition and synergistic therapeutic effect is achieved in KBxN serum transfer arthritis and CIA (32). PTPRS is highly expressed in human RA synovial fibroblasts. Fc-IgI&2 inhibits RA synovial fibroblasts motility *in vitro* (32). All these results suggest that manipulation of PG switch between PTPRS and PG can be potentially applied for treatment of human RA and may be combined with TNF inhibition.

PG switch is in operation in central nervous system. PTPRS is expressed by neurons. Binding of PTPRS to HS-containing PG causes oligomerization and functionally inactivation for axonal extension. In contrast, binding of PTPRS to CS-containing PG inhibits axonal growth (65). Moreover, inhibition of PTPRS can induce neuronal regeneration in spinal cord contusion models (67, 68). These results suggest that strategies in inhibition of PTPRS is therapeutic for arthritis and in favor of neuronal regeneration. We hope that the same beneficial effects of PTPRS inhibition will also be achieved in treating human RA.

## Interrupting Intercellular Signaling for Modulation of Fibroblasts

In RA synovium, the expanded fibroblasts in the lining and sublining layers reside in different anatomical regions and execute related but different activities (29), and phenotypically are distinct subpopulations. However, they are likely derived from same origin. Perivascular fibroblasts receive signals from vascular endothelial cells to proliferate and differentiate and migrate to lining layer. This process is evident by gradient increase of PRG4 but decrease of Thy1 expression from sublining to lining fibroblasts (24). Among the signals conduced between endothelial cells and fibroblasts, NOTCH signaling, particularly NOTCH3 is critical. NOTCH3 is highly expressed by perivascular fibroblasts. In an organoid culture system, silencing NOTCH3 gene expression by siRNA diminished endothelium induced fibroblast expansion and gradient cell surface Thy1 and PRG4 expression. The critical role of NOTCH signaling has been tested in animal models of arthritis. In a rat CIA model, a NOTCH1 and NOTCH3 inhibitor (LY411575) showed therapeutic effect of arthritis, presumably by inducing fibroblast death since LY411575 is able to induce death of human synovium derived fibroblast cell line, MH7A *in vitro* (69). Using monoclonal antibodies, Wei et al. (24) demonstrated that anti-NOTCH3 monoclonal antibody has profound therapeutic effect in arthritis compared with moderate effect by monoclonal antibody to NOTCH1 (Figure 2B). Furthermore, NOTCH3 knockout mice had diminished arthritis induced by KBxN serum transfer compared with wild type animals (24). NOTCH3 gene deleted mice develop a normal joint structure suggests that NOTCH3 targeted therapy is safe for treating arthritis (24, 26). Further development for RA therapy by targeting NOTCH3 signaling is warrant.

## Targeting Synovial Fibroblast Proliferation and Invasion

Several modalities with various mechanisms of action are able to inhibit proliferation, migration and/or invasion of RA synovial fibroblasts (Figure 2C) and have been investigated for development of fibroblast targeted therapy.

### Yes-Associated Protein and Transcriptional Co-activator With PDZ-binding Motif

Yes-associated protein (YAP) and transcriptional co-activator with PDZ-binding motif (TAZ) are transcriptional co-activators involving in regulation of cell growth and differentiation during development and neoplastic progression (70). YAP and TAZ share structure similarity and have overlapped functions; both exert their transcriptional activity via translocation to the nucleus and by interaction with transcriptional enhanced associate domains (TEAD). YAP/TAZ transcriptional activity is enhanced in RA fibroblasts to promote their proliferation and invasive behavior (71, 72). *In vitro*, blocking YAP/TAZ activity using verteporfin, which inhibits YAP/TAZ binding to TEAD, resulted in reduced RA fibroblast resistance to apoptosis, diminished proliferation, less invasion, and poor inflammatory response (72). Moreover, in organoid culture system, verteporfin was able to prevent RA fibroblasts to form synovial lining layer and interrupt already formed lining layer. This effect was the result of YAP/TAZ mediated c-Jun nuclei translocation that was suppressed by verteporfin. Further, in an adjuvant induced arthritis (AIA) model, administered before arthritis onset, verteporfin reduced arthritis severity although arthritis was not prevented. After onset of arthritis, verteporfin was able to block further progression of arthritis (71). In another study, YAP was found to form a complex with PTPN14 to recruit SMAD3 to nucleus of RA synovial fibroblasts to induce the aggressive behavior. Verteporfin decreased RA synovial fibroblast invasion into cartilage *in vivo* in a severe combined immunodeficiency mouse model and reduced severity of KBxN serum transferred arthritis (71). All these data indicate that YAP/TAZ contributes to the invasive behavior of RA synovial fibroblasts and interrupting YAP/TAZ transcriptional activity is a viable strategy to explore as an alternative therapy for RA.

### Seliciclib—Cell Cycle Dependent Kinase Inhibitor

Cell cycle dependent kinase (CDK) inhibitor, seliciclib is under development for cancer therapy (73). Seliciclib suppresses synovial fibroblast proliferation by inhibiting CDK2 and induction of endogenous CDK inhibitor p21 which was shown to be down regulated in synovial fibroblasts of RA patients (74, 75). In addition, independent of cell cycle inhibition, seliciclib was shown to inhibit expression of collagen, fibronectin and connective tissue growth factor in normal and scleroderma fibroblasts (76). In a KBxN serum transfer arthritis model, injection of seliciclib significantly reduced the severity of arthritis (77) although the therapeutic effect of seliciclib was not directly attributed to inhibition of fibroblasts. Based on these findings, a phase Ib clinical trial was conducted in 15 RA patients who display active disease despite treatment with TNFi either as monotherapy or in combination with other disease modifying anti-rheumatic drugs (DMARD) (78). The trial was designed to assess safety of seliciclib in treating RA patients. The maximum tolerable dose is 400 mg/day; the safety profile is acceptable for future efficacy trial. At 4 weeks, 9 patients showed reduction of DAS28-CRP score although these may represent regression to the mean. Nevertheless, this finding indicates further evaluation of seliciclib for RA treatment, especially in combination with other DMARDs including TNFi is warrant. Seliciclib is not myelossuppressive, therefore, exceeding immunosuppression is not expected in combination of seliciclib with other DMARDs.

### Epigallocatechin-3-Gallate

Epigallocatechin-3-gallate (EGCG), a compound derived from green tea, displays potent antioxidant, anti-inflammatory, and antioncogenic activity (79); and has been shown to be able to ameliorate arthritis in animal models (80, 81). Effects of EGCG on RA synovial fibroblasts are multifold. In the *in vitro* culture system, EGCG does not directly cause cytotoxicity of RA synovial fibroblasts, but promotes apoptosis in TNF sensitized cells by blocking myeloid cell leukemia 1 expression (82); inhibits IL-1β induced chemokine production and MMP2 activation (83); and inhibits IL-6 synthesis and suppresses IL-6 trans-signaling by inducing production of soluble gp130 production (80). It is required to determine whether the *in vivo* anti-arthritic effect of EGCG takes place by selectively targeting on synovial fibroblasts but not affecting other effector cells in arthritis. It has been shown that in IL-1 receptor antagonist knock mouse arthritis and CIA models, EGCG attenuates arthritis by inhibiting STAT3 and hypoxia induced factor-α which leads to reduction of Th17 cells (84, 85). The effects of EGCG on other cell types indicates its wide spectrum of antiinflammatory effect and may not be a viable candidate for fibroblast targeted therapy.

### Chinese Herbal Extracts

Similarly, extracts from Chinese herbal drugs such as kirenol (86), piperlongumine (87) and 3,3′-diidolylmethane (88) have been shown *in vitro* to inhibit RA synovial fibroblast proliferation, migration, and invasion. In addition, they were able to suppress MMP production by RA fibroblasts. Moreover, kirenol and 3,3′-diidolylmethane are able to attenuate CIA and AIA, respectively (86, 88). Further investigations are required to delineate whether the effects of these drugs are specific to fibroblasts.

## Promotion of Cellular Senescence of Fibroblasts

Promotion of cellular senescence has been proposed and actively explored as an approach to cancer therapy (89, 90). RA synovial fibroblasts display some features of tumor cells (4), thereby, pro-senescence in these fibroblasts is potentially therapeutic for RA (Figure 2D). Indeed, Montero-Melendez et al. (91) demonstrated that induction of synovial fibroblast senescence via activation of a G-protein coupled receptor, melanocortin type 1 receptor (MC1R) can ameliorate an experimental arthritis. MC1R is highly expressed by RA fibroblasts. Activation of MC1R using a selective agonist, BMS-470539, but not a non-selective agonist, induced senescence in RA fibroblasts. The specific role of MC1R in this senescence was further confirmed in primary mouse synovial fibroblasts that *MC1R* knockout abrogate the effect of BMS-470539. Furthermore, this MC1R activation induced fibroblast senescence involves phosphorylation of extracellular signal-regulated protein kinase (ERK) 1/2, a mitogen-activated protein kinase (MAPK) family protein, which have been shown to promote cellular senescence (92). Importantly, BMS-470539 was able to suppress KBxN serum transfer induced arthritis in mice and the therapeutic effect was associated with *in vivo* synovial fibroblast senescence. The therapeutic effect of BMS-470539 was countered by senolytic drugs (91).

These results are encouraging although further confirmatory evaluation in chronic arthritis models would be required for long-term efficacy and possible adverse effects associated with pro-senescence. Senescence of joint cells, especially chondrocytes is increased in osteoarthritis (OA) (93). It is to be determined if therapy with pro-senescence in fibroblasts and off-targeted senescence of other joint cell types will result in OA of the joint. Another concern associated with pro-senescence as a therapy is the removal of senescent cells from the tissue. Insufficient elimination of the senescent cells after induction of pro-senescence might be detrimental and senolysis may be required. Interestingly, local clearance of senescent cells is beneficial in post-traumatic OA model (94).

## Induction of Fibroblast Apoptosis

RA synovial fibroblasts show reduced rate of apoptosis. Thereby, inducing cell death is an attractive approach to ablate synovial fibroblasts for RA therapy (Figure 2E).

### p53 Upregulated Modulator of Apoptosis

Somatic mutation of tumor suppressor gene, *TP53* (encoding a protein commonly called p53) is one of the important mechanisms responsible for insufficient apoptosis and invasiveness of RA fibroblasts (95, 96). Thereby, promoting apoptosis of fibroblasts is a plausible strategy for RA therapy. Along this line, Firestein and colleagues first demonstrated that *PUMA* (p53 upregulated modulator of apoptosis) gene expression is deficient in RA fibroblasts (97); subsequently, adenovirus vector transfection of *PUMA* resulted in rapid apoptosis of fibroblasts with the activation of caspase 3 (97). Interestingly, *PUMA* induced apoptosis is independent of p53 expression (98), which is of significance in practice for developing a therapy since induction of *PUMA* expression alone without presence of active p53 is sufficient to induce death of these fibroblasts in the synovium. However, gene therapy with *PUMA* had been hampered by poor gene delivery efficiency in fibroblasts until recently. This problem was circumvented by Hong et al. (99) by conjugating human adenovirus type 5 (HAdV5) to a baculovirus vector expressing the Coxsackie-adenovirus receptor (CAR) on its envelope. The modified BV^CAR^-HAdV5 vector efficiently delivered *PUMA* gene (BV^CAR^-HAdV5-PUMA) into RA synovial fibroblasts and induced rapid cell death *in vitro*. Furthermore, in a rat AIA model, single intraarticular injection of BV^CAR^-HAdV5-PUMA significantly reduced inflammation, improved joint function, and decreased joint erosion and bone loss. HAdV5 is a biological safe virus vector for gene therapy, but the intraarticular delivery limited its further development as a viable therapeutic strategy since RA patients require systemic treatment.

### Cadmium

Metal element, cadmium has p53-dependent pro-apoptotic properties (100) and has been tested for treating arthritis in animal models (101). Cadmium was able to induce apoptotic cell death of synovial fibroblasts isolated from patients with RA. Intraarticular injection of cadmium in rats with AIA can suppress inflammation. Although this proof of concept study demonstrated the anti-arthritic effect of cadmium, several issues including toxicity, association of cadmium with an increased risk of RA (102, 103), and local administration will limit its use as a therapeutic for RA.

## Inhibition of Glucose Metabolism of Fibroblasts

The tumor-like behaviors of RA fibroblasts such as activation, migration and invasion are associated with increased cell metabolism. There is ample evidence to indicate that glucose metabolism plays an important role. Therefore, interruption of glucose metabolism of fibroblasts is a plausible approach to novel therapy of RA. As has been demonstrated in several murine models of RA, inhibition of glycolysis can significantly reduce severity of arthritis (104, 105). However, global inhibition of glycolysis is not desirable as a therapy. Fibroblast specific inhibitor of glucose metabolism is required. Among all the glycolytic enzymes involved, hexokinase 2 (HK2) may be a relatively selective target (Figure 2F). HK2 is an inducible isoform that is selectively highly expressed in skeletal and cardiac muscles and adipose tissue (106), and is highly expressed in RA but little in OA synovial tissue (107). Silencing HK2 resulted in reduction of migration and invasion of RA fibroblasts *in vitro*. Ablation of HK2 significantly reduced severity of arthritis and bone and cartilage damage in KBxN serum transfer induced arthritis (107).

Currently, a HK2 specific inhibitor is not available, but a glucose analog, 2-deoxy-D-glucose (2-DG) has been extensively evaluated as an agent for inducing cancer cell death and cancer therapy (108). Administration of 2-DG reduced the severity of spontaneous arthritis in KBxN mice. However, the therapeutic effect of 2-DG was attributed to inhibition of glucose metabolism in T follicular helper (Tfh) cells. The effect of 2-DG on fibroblast activity remains to be possible, but unfortunately this was not investigated in this work (109). This result on T cells is in consistence with the previous findings that loss of HK2 mildly reduced colitis in interleukin-10 deficient mice and ovalbumin induced airway inflammation (110). Furthermore, HK2 is not essentially required for T cell glucose metabolism *in vitro* and loss of HK2 did not impair clearance of lymphocytic choriomeningitis virus infection, suggesting inhibiting HK2 activity would have less immune compromise (110). Further studies are required to clarify the targeted cell types of 2-DG in RA models and a fibroblast specific HK2 inhibition strategy is required to develop.

## JAK Inhibitors for Fibroblast Targeted Therapy

Janus kinase (JAK) inhibitors are approved therapy for RA. JAK-signal transducer and activator of transcription (STAT) pathway is active in RA synovial fibroblasts in response to cytokine stimulation (111). As expected, JAK inhibitors are able to block cytokine mediated inflammatory production by fibroblasts; these include stimulation by cytokines such as oncostatin M, IL-6, TNF, IL-1, and interferon-γ (111, 112).

Current JAK inhibitors are not fibroblast specific. Among the JAK inhibitors, peficitinib was able to suppress synovial fibroblast migration *in vitro* and may induce fibroblast apoptosis suggesting that peficitinib may have advantage over other JAK inhibitors on targeting fibroblasts (Figure 2G) (113, 114). Since high dose of peficitinib is tolerated, it may in particular be useful for treating RA patients with pauci-immune pathotype which is poorly responsive to conventional DMARDs or TNF inhibitors (115). Selective JAK inhibitors for JAK-STAT pathway in fibroblasts are to be uncovered for this purpose. On the other hand, synovial fibroblasts are major source of IL-6 production in RA joint (25, 116). Moreover, IL-6 acts in an autocrine amplification mechanism on activation of fibroblasts (116). Therefore, JAK inhibitors with profound activity to block IL-6 signaling may have profound impact on fibroblast activation in RA.

## Anti-fibrotic Drugs

Pirfenidone [5-methyl-1-phenyl-2-(1H)-pyridone], one of the heterocycle pyridones, is approved for treating idiopathic pulmonary fibrosis. Pirfenidone was first developed for anti-pyretic and analgesic use, but was found to have anti-fibrotic effect. Pirfenidone has pleiotropic effects including inhibition of transforming growth factor (TGF)-β-mediated proliferation of fibroblasts. In cultured synovial fibroblasts, pirfenidone can reduce the expression of intercellular adhesion molecule-1. Pirfenidone can also suppress TGF-β induced collagen type I production and inhibit myofibroblasts activity to reduce extracellular matrix deposition (117). In a rat CIA model, pirfenidone was shown to ameliorate arthritis and reduction of MMP3 and vascular endothelial growth factor (VEGF) expression in the joint (118). In Simian Vacuolating Virus 40 large T antigen transformed human synovial fibroblast cell line, MH7A cells, pirfenidone significantly reduced TNF-induced production of inflammatory cytokines, VEGF, and MMPs. Moreover, pirfenidone significantly inhibited VEGF by vascular endothelial cell line, EA.hy926 cells (118). Further studies are required to delineate whether these effects of pirfenidone fibroblast selective.

Another anti-fibrotic drug, nintedanib, a potent tyrosin kinase inhibitor that inhibits growth factor stimulated migration and proliferation of fibroblasts and TGF-β-induced transformation to myofibroblasts. In a zymosan-induced arthritis and pulmonary fibrosis model in SKG mice, treatment at early stage of disease, nintedanib was able to significantly reduce severity of arthritis but has no effect on progression of lung fibrosis. In contrast, treatment started when pulmonary fibrosis established, nintedanib was able to reduce pulmonary fibrosis but had no effect on established arthritis (119). Nintedanib is approved for treating idiopathic pulmonary fibrosis. There are case reports in the literature that nintedanib has been tried in RA related interstitial lung disease and showed benefit (120). It would be interesting to investigate further how ninetedanib will affect synovial fibroblasts in RA patients and whether ninetedanib will show anti-arthritic effect.

## Concluding Remarks

RA synovial fibroblasts are valid therapeutic target candidates and current strategies for intervention have been explored (Table 1 and Figure 2). Especially, fibroblasts are not professional immune cells for host defense although they display features of inflammatory cells and can present self-antigens during the disease process of RA. Therapies directed at suppression of the function or ablation of synovial fibroblasts will be less likely to cause immunosuppression. Therefore, development of fibroblast targeted therapy will be particularly practical for combination therapy with currently available immune component directed therapies. Another concern with regard to fibroblast ablation therapy is impairment of wound healing since fibroblasts are critical for tissue repair. However, in preclinical tumor models, ablation of fibroblasts did not negatively affect wound healing although these need to be further evaluated in human studies (51, 52, 59).

**Table 1:** Agents for fibroblast targeted therapy for RA^*^.

**Agent**	**Target and mechanism of action**	**Developing stage**	**References**
	**Cell surface protein as target**		
Monoclonal antibodies	CDH-11: inhibit formation of synovial lining layer	Phase II (ineffective)	(34)
Monoclonal antibodies	FAP: ablation of fibroblasts	Pre-clinical	(49, 50)
Fc-Ig1&2	PTPRS: decoy receptor to inhibit PTPRS binding to proteoglycan and block fibroblast invasion	Pre-clinical	(31, 32)
	**Interruption of intercellular signaling**		
Monoclonal antibodies	NOTCH3: blocking NOTCH3 signaling to inhibit fibroblast differentiation	Pre-clinical	(24)
	**Inhibition of fibroblast proliferation and invasion**		
Verteporfin	YAP/TAZ: blocks YAP/TAZ binding to TEAD to inhibit fibroblast proliferation and invasion	Pre-clinical	(71, 72)
Seliciclib	CDK2: blocks kinase activity to inhibit fibroblast proliferation	Phase I	(78)
EGCG	Pleotropic: anti-inflammatory, anti-oxidant and anti-oncogenic. Inhibits fibroblast proliferation	Pre-clinical	(80, 81)
Chinese herbal extracts	Likely pleotropic: inhibit fibroblast proliferation and invasion	Pre-clinical	(86, 88)
	**Induction of cellular senescence**		
BMS-470539	MC1R: agonist, activates MC1R to induce fibroblast senescence	Pre-clinical	(91)
	**Promotion of apoptosis**		
BV^CAR^-HAdV5-PUMA	PUMA: virus mediated gene delivery to induce fibroblast apoptosis	Pre-clinical	(99)
Cadmium	Metal element: induction of fibroblast apoptosis	Pre-clinical	(101)
	**Inhibition of glucose metabolism**		
2-deoxy-D-glucose (2-DG)	HK2: non-HK2 selective inhibitor, inhibit fibroblast glucose metabolism	Pre-clinical	(109)
	**JAK inhibitor and anti-fibrotic drugs**		
Peficitinib and others	JAK: inhibits JAK activity, non-selective. Approved clinical treatment of RA, but fibroblast selective JAK inhibitors are to uncover		(113, 114)
Pirfenidone	Anti-fibrotic: approved for treating idiopathic pulmonary fibrosis	Pre-clinical (repurpose for treating RA)	(118)
Nintedanib	Anti-fibrotic: approved for treating idiopathic pulmonary fibrosis	Pre-clinical (repurpose for treating RA)	(119)

* *RA, rheumatoid arthritis; CDH-11, cadherin-11; FAP, fibroblast activation protein; Fc-Ig1&2, IgG Fc-immunoglobulin-like domains 1&2 fusion protein; PTPRS, protein tyrosine phosphatase receptor sigma; NOTCH3, neurogenic locus notch homolog protein 3; YAP/TAZ, Yes-associated protein (YAP) and transcriptional co-activator with PDZ-binding motif (TAZ); TEAD, transcriptional enhanced associate domains; CDK2, cell cycle dependent kinase 2; EGCG, epigallocatechin-3-gallate; MC1R, melanocortin type 1 receptor; PUMA, p53 upregulated modulator of apoptosis; BV^CAR^-HAdV5-PUMA, human adenovirus type 5 (HAdV5) to a baculovirus vector expressing the Coxsackie-adenovirus receptor (CAR); HK2, hexokinase 2; JAK, Janus kinase*.

In addition to the strategies discussed above, mitogen-activated protein kinase (MAPK) are highly activated in RA synovial fibroblasts. However, targeting MAPK pathway for therapy has not be successful in clinical trials (discussed in details by Nygaard and Firestein (12). Also, targeting the imprinted signature of RA synovial fibroblasts is another attractive approach. For example, modulation of histone-modifying enzymes may lead to remodeling and restoration of the homeostasis of RA synovial fibroblasts [reviewed by (12)].

Clinically, RA patients with pauci-immune synovial pathotypes respond poorly to current DMARDs which are mainly directed to immune suppression. Presumably fibroblasts are the predominant effector cell types mediating the disease process in these patients (8, 115). This RA subpopulation will better serve for testing efficacy of fibroblast targeted therapies in clinical studies. Peficitinib, one of the newer JAK inhibitors was shown to preferentially act on JAK-STAT pathway in RA synovial fibroblasts and hence would be a good candidate to try on this RA subpopulation (113, 114).

## Author Contributions

The author confirms being the sole contributor of this work and has approved it for publication.

## Funding

C-QC's work was supported by an Innovative Award from American College of Rheumatology Research Foundation and by a VA Merit Review grant (I01BX005195).

## Conflict of Interest

The author declares that the research was conducted in the absence of any commercial or financial relationships that could be construed as a potential conflict of interest.

## Publisher's Note

All claims expressed in this article are solely those of the authors and do not necessarily represent those of their affiliated organizations, or those of the publisher, the editors and the reviewers. Any product that may be evaluated in this article, or claim that may be made by its manufacturer, is not guaranteed or endorsed by the publisher.
